# Advances in the Synthesis of Covalent Triazine Frameworks

**DOI:** 10.1021/acsomega.2c06961

**Published:** 2023-01-23

**Authors:** Longfei Liao, Mingyu Li, Yongli Yin, Jian Chen, Qitong Zhong, Ruixing Du, Shuilian Liu, Yiming He, Weijie Fu, Feng Zeng

**Affiliations:** † School of Materials Science and Engineering, Harbin Institute of Technology (Shenzhen), Shenzhen 518055, Guangdong, China; ‡ Space Science and Technology Institute (Shenzhen), Shenzhen 518117, Guangdong, China; § State Key Laboratory of Materials-Oriented Chemical Engineering, College of Chemical Engineering, Nanjing Tech University, Nanjing 211816, Jiangsu, China

## Abstract

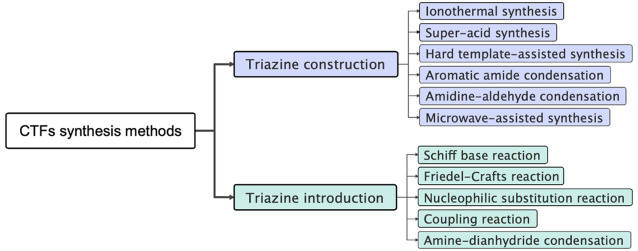

Covalent triazine frameworks (CTFs) are a class of organic polymer materials constructed by aromatic 1,3,5-triazine rings with planar π-conjugation properties. CTFs are highly stable and porous with N atoms in the frameworks, possessing semiconductive properties; thus they are widely used in gas adsorption and separation as well as catalysis. The properties of CTFs strongly depend on the type of monomers and the synthesis process. Synthesis methods including ionothermal polymerization, amino-aldehyde synthesis, trifluoromethanesulfonic acid catalyzed synthesis, and aldehyde–amidine condensation have been intensively studied in recent years. In this review, we discuss the recent advances and future developments of CTFs synthesis.

## 1 Introduction

**Figure 1:**
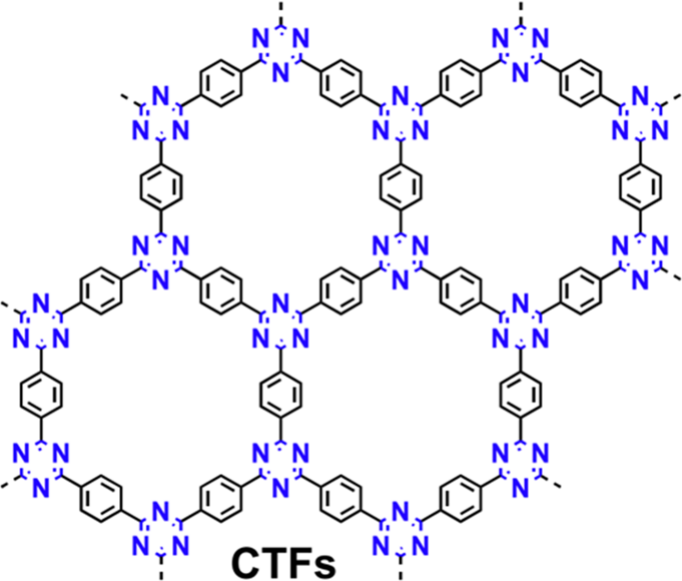
CTFs constructed by aromatic 1,3,5-triazine rings.

Covalent triazine frameworks (CTFs) are a type of organic polymers, constructed by aromatic 1,3,5-triazine rings (Figure 1) with planar π-conjugation properties.^1−7^ The conjugation between aromatic rings and triazine rings reduces the total energy of π-conjugated molecules in the frameworks, thus improving the chemical stability.^3,4,8,9^ Moreover, N-containing CTFs frameworks are generally porous and semiconductive and are promising for application in adsorption/separation and catalysis.

The CTFs generally possess large specific surface areas, and the N atoms in the frameworks provide sites to anchor molecules; thus they are suitable materials for the adsorption of gases and organic compounds.^10−17^ Accordingly, they have been used for CO_2_ capture and separation.^18^ Zhong et al. synthesized porous CTFs with a large number of microporous and ultramicroporous structures by ionothermal polymerization. The obtained CTFs perform well for CO_2_ capture.^19^ Moreover, Cooper et al. synthesized 1,3,5-triazine node conjugated microporous polymers (TCMPs) via Pd-catalyzed Sonogashira cross-coupling.^20^ Although the surface areas of TCMPs are similar to those of the corresponding benzene-linked conjugated microporous polymers, TCMPs exhibit a higher CO_2_ capture capacity (i.e., 1.45 mmol g^–1^, 298 K, 1 bar), due to the presence of triazine groups.

Besides, Wang et al. synthesized CTFs by ionothermal polymerization to adsorb and remove organic dyes in an aqueous solution.^10^ They found that CTFs’ adsorption capacity for Rhodamine B is about 3 times that of activated carbon. Also, CTFs possess high adsorption capacities for reactive brilliant red X-3B and direct acid-resistant scarlet 4BS.^10^

Moreover, CTFs possess a low framework density and micropore-dominated pore structures and thus can be applied for gas storage.^21−25^ Thomas et al. investigated H_2_ storage in a series of CTFs. Among them, CTFs synthesized from 4,4′-biphenylcarbonitrile (DCBP) with a specific surface area of 2475 m^2^ g^–1^ store 1.55 wt % H_2_ at 1 bar and 77 K.^26^ Besides, Giambastiani et al. modified the ionothermal polymerization method to synthesize pyridine-functionalized CTF-py^HT^.^27^ CTF-py^HT^ with a specific surface area of 3040 mg^2^ g^–1^ exhibits a very high H_2_ uptake (2.63 wt %, 1 bar, 77 K; 4.53 wt %, 20 bar, 77 K).

Due to CTFs’ fully covalent structure, as well as their high thermal and chemical stabilities, they are ideal materials as catalyst carriers for liquid-phase reactions.^28,29^ The presence of triazine units and N-heterocyclic groups leads to CTFs’ high N content, which promotes many catalytic reactions. For example, the electron-donating properties of N increase the number of delocalized electrons in the carbon frameworks, thereby promoting the active site for electrochemical oxygen reduction.^30−34^ Also, CTFs have been developed as capacitive electrode materials for supercapacitor research due to their high N content, porous structure, large specific surface area, and low resistivity.^35,36^ Besides, CTFs can “immobilize” transition metal complexes by anchoring on N-containing functional groups (e.g., amine, pyridine, and triazine groups), thus inhibiting metal agglomeration and subsequent deactivation.^37−39^

Since CTFs have a structure (i.e., triazine ring) similar to g-C_3_N_4_, they are potential metal-free polymer photocatalysts.^40−47^ According to first-principles calculations, the highest occupied molecular orbital (HOMO) and lowest unoccupied molecular orbital (LUMO) of CTF-1 are −0.5 eV and 2.0 eV, respectively, providing enough driving force for the splitting of water, whose equilibrium voltage is 1.23 V.^48^

**Figure 2:**
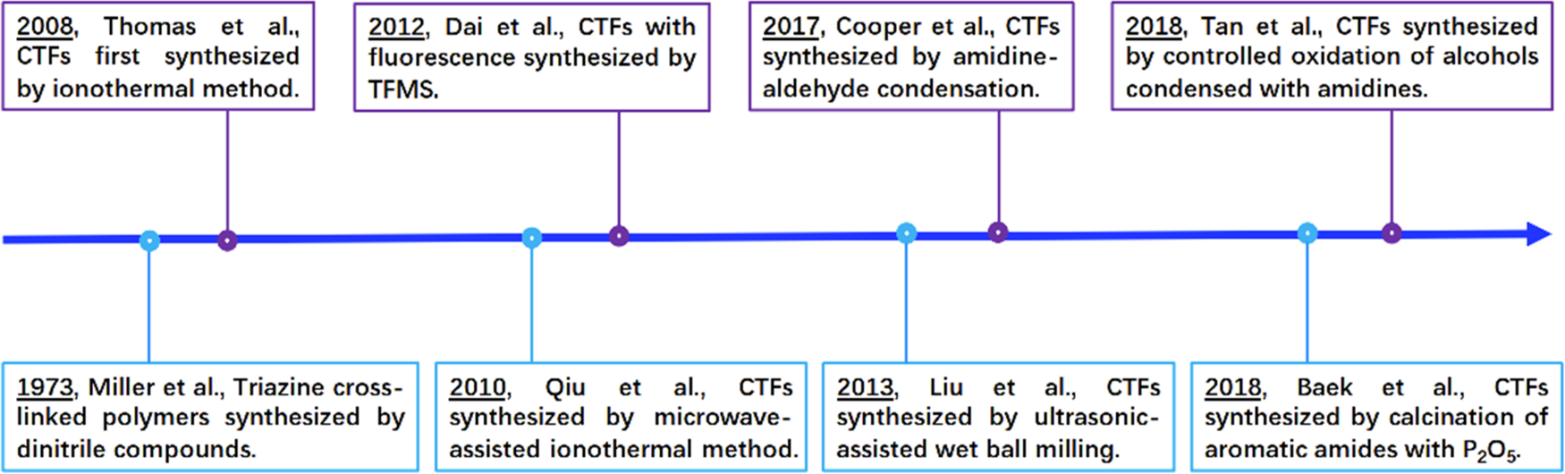
Development of CTFs synthesis.

CTFs’ structure highly depends on the synthesis process; thus tailoring the monomers, synthesis methods, and synthesis conditions of CTFs is of great importance for controlling the structure. CTFs were first synthesized by the polymerization of a single type of monomer, and then later CTFs were synthesized by the polymerization of two or three types of monomers. Up to now, a variety of synthetic routes for CTFs have been developed, and the synthesis conditions have developed from harsh conditions (i.e., high temperature and O_2_ free) to today’s mild open systems (i.e., room temperature and atmospheric environment). As early as 1973, Miller from Texaco Inc. obtained highly stable cross-linked polymers based on triazine structural elements using dinitrile compounds derived triazine as the substrate; however, this did not attract scientists’ attention during that time.^21^ It was not until 2008 that Kuhn, Antonietti, and Thomas synthesized CTFs by ionothermal polymerization using terephthalonitrile and proposed the scientific concept of “CTFs” for the first time.^26^ Since then, typical methods for CTFs synthesis including ionothermal polymerization, microwave-assisted ionothermal synthesis, amino-aldehyde synthesis, trifluoromethanesulfonic acid catalyzed synthesis, and aldehyde–amidine condensation have been developed (Figure 2).

## 2 Design and Synthesis of CTFs

CTFs are constructed by triazine units with various specific functional groups. The monomers’ properties and the type of bonding between them significantly influence the CTFs’ structure and properties such as crystallinity, surface area, porosity, light absorption ability, and chemical stability. For example, CTFs connected by superstrong covalent bonds are mostly amorphous or semicrystalline, because in a highly dynamic polymerization process the stronger the covalent bond, the more difficult it is to form an ordered structure.^49^ Thus, the types of monomers and the polymerization process are of great importance in CTFs synthesis.

**Figure 3:**
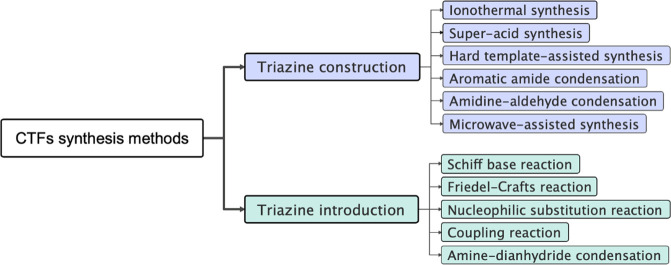
Representative CTFs synthesis methods.

Since the triazine unit is the core functional group that contributes to the excellent properties and wide range of applications of CTFs, the synthesis methods of CTFs are classified into two categories based on the way the triazine unit is incorporated, i.e., synthesis of CTFs by constructing triazine units and synthesis of CTFs by directly introducing triazine unit containing monomers (Figure 3). Synthesis of CTFs by constructing triazine units includes methods such as ionothermal synthesis, superacid synthesis, hard-template-assisted synthesis, aromatic amide condensation, and microwave-assisted synthesis, while synthesis of CTFs by directly introducing triazine unit containing monomers includes methods based on the Schiff base reaction, Friedel–Crafts reaction, nucleophilic substitution reaction, coupling reaction, and amine–dianhydride condensation. Notably, the polymers connected by triazine rings are called CTFs, while those containing triazine structures but linked by other bonds are called triazine polymers (e.g., triazine covalent organic frameworks). However, due to the similarities in the properties and synthesis of these two materials, we discuss both of them in this review.

### 2.1 Synthesis of CTFs by Constructing Triazine Units

**Figure 4:**
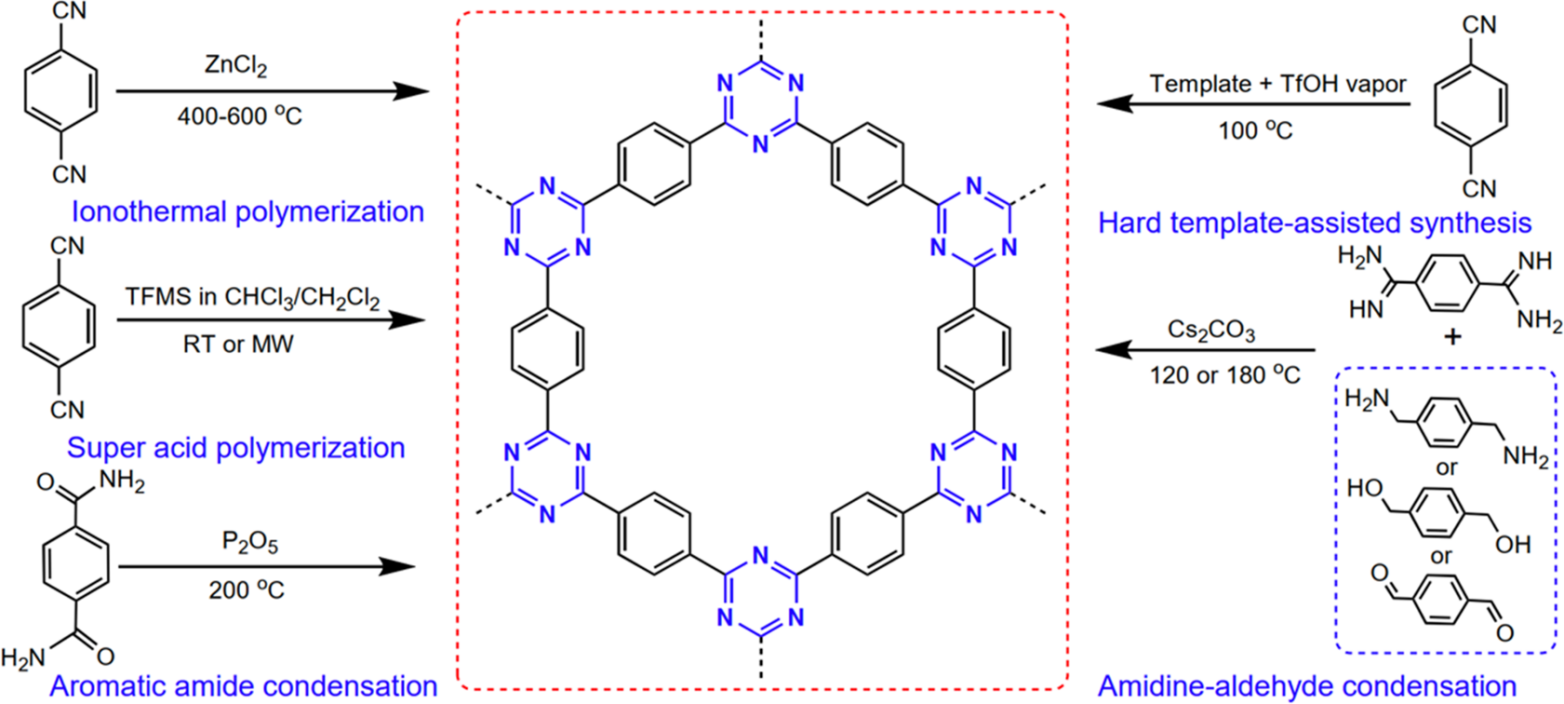
Typical synthesis of CTFs through the construction of triazine units.

Triazine units are generally constructed by polymerization based on electron-absorbing effects using monomers containing nitrile, amide, or amidine groups. As presented in Figure 4, the polymerization generally occurs through nitrile-based trimerization reactions (e.g., ionothermal polymerization,^26^ superacid-catalyzed polymerization,^50^ and hard-template-assisted synthesis^51^), aromatic amide condensation,^52^ and amidine–aldehyde condensation,^46^ which will be discussed in detail in sections 2.1.1–2.1.5.

#### 2.1.1 Ionothermal Polymerization

Generally, the ionothermal polymerization of nitrile monomers occurs through Lewis acid–base interactions. Nitrile-based monomers first dissolve in the high-temperature molten ZnCl_2_ and then undergo reversible dynamic trimerization catalyzed by ZnCl_2_. Thomas’s group first synthesized CTF-1 by the ionothermal polymerization of *p*-benzenedicarbonitrile monomer at 400 °C with molten ZnCl_2_ as the catalyst and solvent.^26^ The obtained CTF-1 is a highly stable porous material with a specific surface area of 791 m^2^ g^–1^ and pore size of 1.2 nm. The high surface areas of CTFs synthesized by ionothermal polymerization are ascribed to the partial carbonization of CTFs due to the high synthesis temperature and the presence of molten ZnCl_2_ as a template.^53^

**Figure 5:**
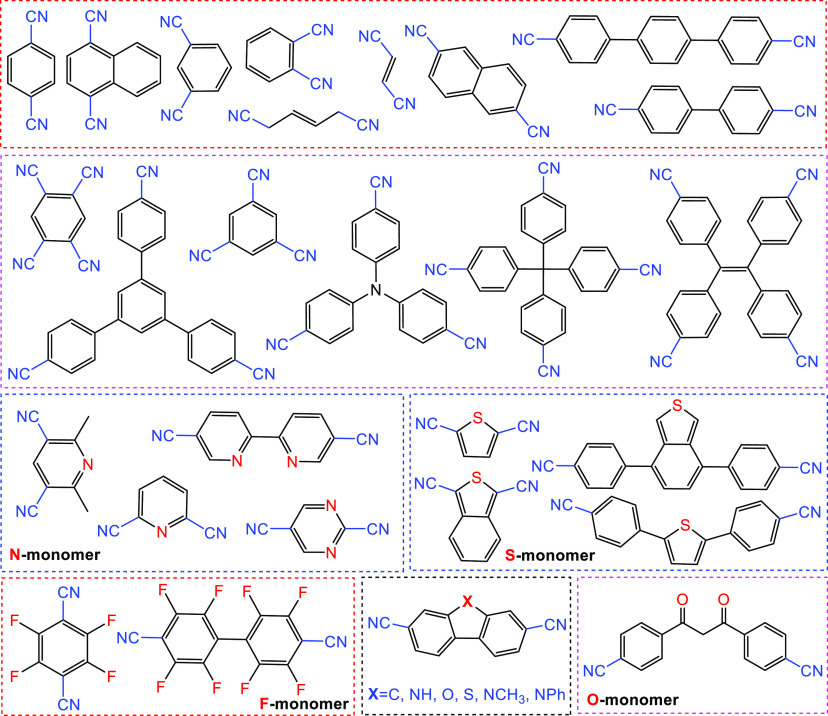
Typical monomers for ionothermal polymerization.

The nitrile monomer is the most important factor influencing the structure and properties of CTFs. Figure 5 presents the typical nitrile-based monomers for ionothermal polymerization. Generally, the monomer possessing a highly symmetrical planar geometry easily forms a regular three-dimensional tubular structure with a large specific surface area. For example, CTF-1-0.1 synthesized by ionothermal polymerization using terephthalonitrile as a monomer achieves a high specific surface area of 1123 cm^2^ g^–1^.^26^ On the contrary, if the monomer itself features a planar and contorted structure, CTFs will be formed with relatively low specific surface areas. The specific surface area of CTF-DCT-0.1 prepared by ionothermal polymerization using 5-dicyanothiophene monomer is 584 cm^2^ g^–1^.^26^

Moreover, introducing monomers containing heteroatoms (e.g., N and F) or specific groups into the structure of CTFs by ionothermal polymerization effectively improves specific properties. The introduction of N-containing functional groups such as amine, imine, and triazine groups increases the basicity of the material and facilitates the stabilization of metal ions, as well as the immobilization of nanoparticles due to the stronger affinity.^54^ Besides, the adsorptive and fluorescent properties of the materials will also be enhanced as the F is incorporated.^15^

ZnCl_2_ as a porogenic agent also affects the porosity and specific surface area of CTFs. During ionothermal polymerization, as the ratio of ZnCl_2_/nitrile-based monomer increases, the long-range-ordered structure is gradually lost, leading to the formation of amorphous but highly porous CTFs.^37^ Temperature is another factor influencing the structure and property of ionothermal-polymerization-obtained CTFs. ZnCl_2_ melts at high temperatures (e.g., ≥400 °C), promoting ionothermal polymerization, but the high temperature is energy consuming and meanwhile destroys the structural integrity leading to partial carbonization. Thus, CTFs synthesized by this method are black powders due to the partial carbonization of the polymers, resulting in uncontrollable positions of the conduction band (CB) and valence band (VB).^55−57^ Accordingly, the utilization of these CTFs in various applications such as photocatalysis is limited.

Since ZnCl_2_ is a good microwave absorber, the synthesis time by ionothermal polymerization can be reduced with the assistance of microwaves, increasing the synthesis efficiency.^58^ In 2010, Zhang et al. used microwave-assisted ionothermal polymerization of terephthalonitrile to synthesize CTFs. The reaction time was greatly reduced from 40 to 1 h.^58^ Even though the reaction time is highly reduced, partial carbonization cannot be avoided due to the high heating rate and high reaction temperature. In 2022, Wang et al.^59^ reported a CTF-based photocatalyst synthesized via an ionothermal method by using a ternary NaCl–KCl–ZnCl_2_ eutectic salt (ES) mixture. The melting point of the ES mixture is approximately 200 °C, which is lower than that of pure ZnCl_2_ (i.e., 318 °C), thus providing milder salt-melt conditions. Accordingly, CTFs prepared by this method overcome high-temperature carbonization. The obtained CTF-ES_200_ (CTF-1 synthesized at 200 °C) exhibits enhanced optical and electronic properties and is efficient for the photocatalytic hydrogen evolution reaction.

Overall, CTFs prepared by ionothermal polymerization with ZnCl_2_ catalyst possess a large specific surface area and high porosity. Accordingly, they show excellent performance in gas adsorption, catalytic carrier, liquid phase adsorption, and electrochemistry. However, the high synthesis temperature leads to irreversible carbonization of CTFs, influencing their optical properties, and is unfavorable for application in fluorescent probe detection and photocatalysis.

#### 2.1.2 Superacid Catalyzed Polymerization

**Figure 6:**
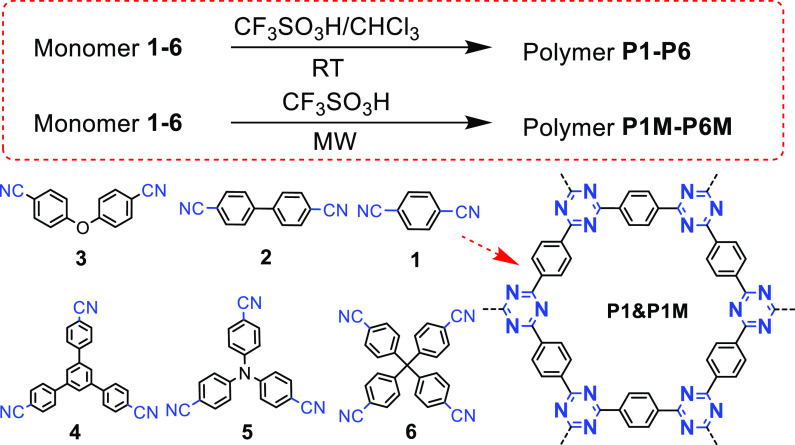
Representative monomers for superacid polymerization. Adapted with permission from ref (50). Copyright 2012 John Wiley and Sons.

To mitigate the carbonization (i.e., formation of black CTFs powder) caused by the high synthesis temperature, in 2012 Cooper et al. developed a microwave-assisted Brønsted acid polymerization method to synthesize CTFs.^50,60^ This method uses trifluoromethanesulfonic acid (TFMS) as a catalyst for the trimerization of aromatic nitrile monomers into CTFs at room temperature with a shortened reaction time due to the assistance of microwaves. CTFs by this method later proved effective for photocatalytic water splitting.^42^ Besides, CTFs were also successfully synthesized using terephthalonitrile as the monomer and trifluoromethanes as the catalyst with either trichloromethane^61^ or dichloromethane^24^ as the solvent. Figure 6 summarizes the representative monomers for superacid catalyzed polymerization, which can be used to synthesize CTFs at room temperature with the assistance of microwaves.

CTFs synthesized by superacid polymerization possess many advantages. First, the low reaction temperature and short reaction time facilitate the synthesis. Second, carbonization of CTFs due to decomposition at high temperatures is avoided, resulting in defect-free frameworks. Third, ZnCl_2_ contamination which possesses photoactivity in the porous materials is eliminated. However, the specific surface area and pore size of CTFs prepared by this method are insufficient for application in adsorption. Besides, these CTFs possess no layered structure and cannot use acid-sensitive building blocks as the substrate. Moreover, due to the strongly corrosive and carcinogenic nature of the catalyst, the synthesis cost is high. Nonetheless, this method retains the optical properties of CTFs, being useful in optics or photocatalysis.

#### 2.1.3 Hard-Template-Assisted Synthesis (or Solid-State Synthesis)

**Figure 7:**
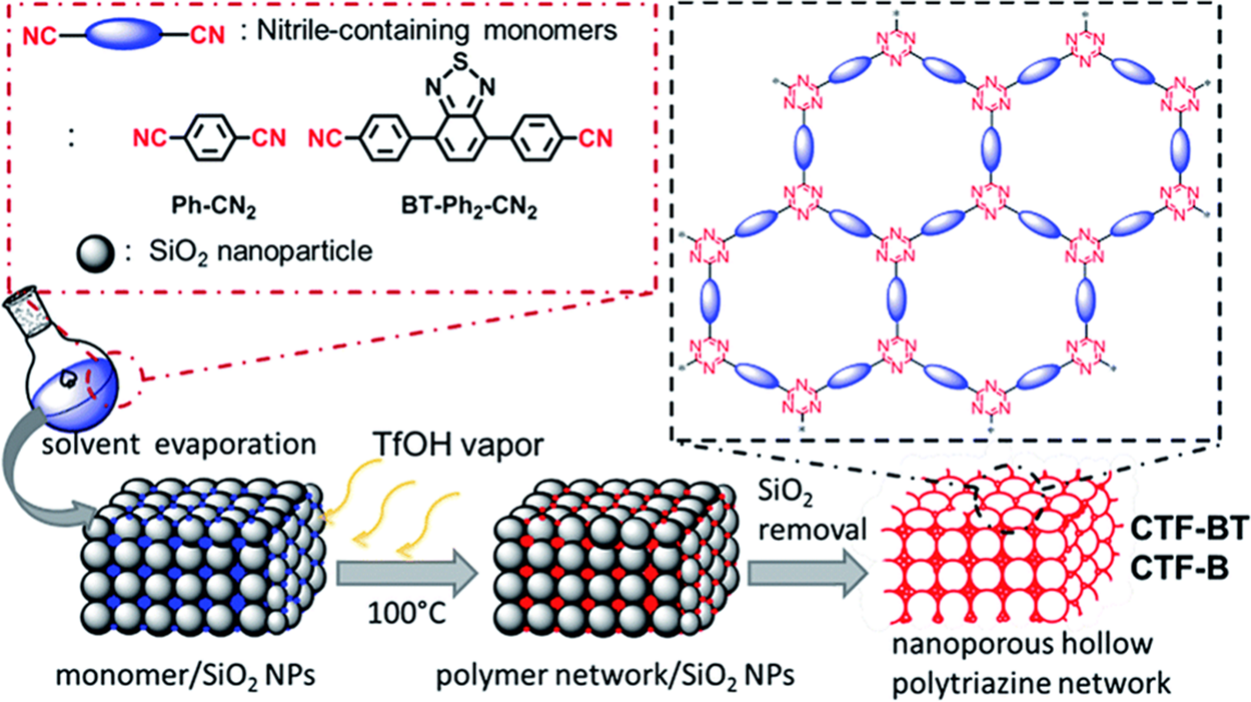
Scheme showing a systematic solid vapor approach for the synthesis of nanoporous hollow polytriazine networks. Reprinted with permission ref (51). Copyright 2016 The Royal Society of Chemistry.

Hollow and porous structures promote mass transfer and light absorption during catalysis. To synthesize hollow and porous CTFs, hard templates have been used to assist the Brønsted acid vapor catalyzed polymerization (Figure 7).^51^ In this process, solutions of nitrile monomers are mixed with uniformly packed SiO_2_ nanoparticles (300 nm in size) which are used as templates. After evaporation of the solvent, polymerization is carried out in a TFMS vapor atmosphere. The SiO_2_ nanoparticles are removed after polymerization to create homogeneous macropores, resulting in hollow nanoporous CTFs with specific surface areas of 90–565 m^2^ g^–1^ and pore volumes of 0.32–0.44 cm^3^ g^–1^. Besides, a similar method uses ordered mesoporous silica SBA-15 as the template and 2,5-dicyanothiophene as the monomer to synthesize a mesoporous CTF-Th@SBA-15 which possesses a specific surface area of 548 m^2^ g^–1^ and a total pore volume of 0.7 cm^3^ g^–1^.^62^ However, as the template is removed, the mesoporous channel collapses, and the specific surface area decreases to 57 m^2^ g^–1^.

#### 2.1.4 Aromatic Amide Condensation

**Figure 8:**
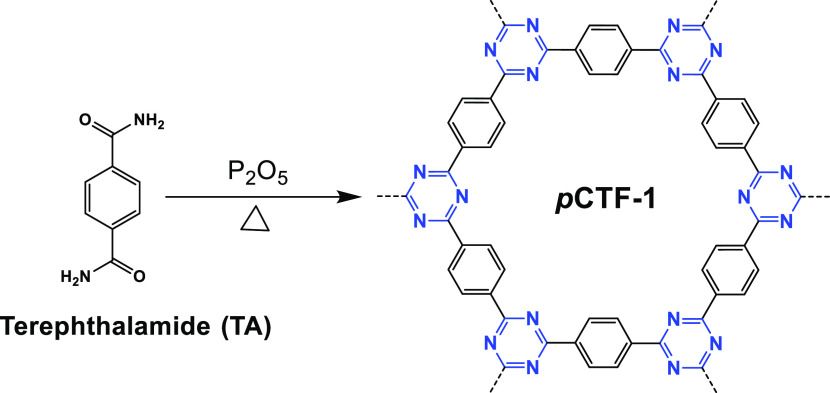
Scheme of *p*CTF-1 synthesis via aromatic amide condensation. Adapted with permission from ref (52). Copyright 2018 John Wiley and Sons.

To avoid using ZnCl_2_ catalyst, which is difficult to completely remove, in 2018 Baek et al. synthesized CTFs by the condensation of aromatic amide derived nitrile compounds using phosphorus pentoxide (P_2_O_5_) as a catalyst and reaction medium (Figure 8).^52^ During the reaction, the aromatic amide group (C(=O)—NH_2_) in terephthalamide is first dehydrated to a nitrile group (C≡N) followed by condensation forming the *s*-triazine rings catalyzed by P_2_O_5_. The *p*CTF-1 prepared by this method possesses a high specific surface area (2034.1 m^2^ g^–1^), good stability, and high crystallinity. Compared to ionothermal or superacid polymerization, this method enables using a wide range of monomers for CTFs synthesis and avoids the presence of ZnCl_2_ contamination in ionothermal polymerization. However, carbonization of the frameworks cannot be avoided.

#### 2.1.5 Amidine–Aldehyde Condensation

**Figure 9:**
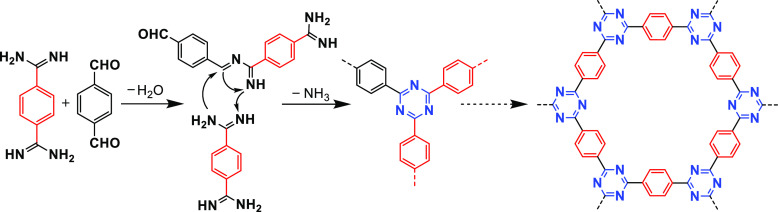
Synthesis of CTFs by amidine–aldehyde condensation. Adapted with permission from ref (46). Copyright 2017 John Wiley and Sons.

To synthesize CTFs on a large scale, as well as avoiding high reaction temperatures or using strong acids in normal synthesis methods, in 2017 Tan et al. synthesized CTFs by a one-pot polycondensation of amidines and aldehydes under mild conditions (Figure 9).^46^ The synthetic condensation of amidine dihydrochloride and aldehyde involves a Schiff base reaction, followed by Michael addition. In a Schiff base reaction, the imine bond is formed due to the dehydration condensation of an aldehyde group and an amino group, while in Michael addition, a C=N bond is formed by the deamination and condensation of the amino group with the imine group, eventually forming a triazine unit. Dimethyl sulfoxide (DMSO) is used as a solvent due to its weak oxidizability and high boiling temperature, and Cs_2_CO_3_ is used as a base due to its proper basicity. This method further expands CTFs’ structural diversity. However, the obtained CTFs are predominantly amorphous owing to the strong aromatic C=N bond.^63^ Due to the relatively low synthesis temperature (i.e., 120 °C) and convenient open synthetic system, the one-pot amidine–aldehyde condensation method can be scaled up to synthesize CTFs at a multigram level.

To further increase the crystallinity of CTFs, Tan et al. developed a strategy to prepare ordered crystalline CTFs via *in situ* oxidation of alcohols followed by amidine–aldehyde polycondensation (Figure 9) based on a new mechanism study, which pointed out that a low nuclei concentration and a slow nucleation process could promote crystallization.^63,64^ In this method, alcohols are first slowly *in situ* oxidized to aldehydes to decrease nucleation rates in DMSO solution. Then, the subsequent polymerization occurs as the temperature reaches the boiling point of DMSO. Polymerization at this high temperature improves the crystallization.^63^ Hence, according to this approach, a series of crystalline CTFs were prepared with high surface areas and good photocatalytic properties. Moreover, this method can be performed in an open system with a mild ensemble temperature (i.e., 120–180 °C), being a potential way for large-scale production.

More recently, Jin et al. synthesized two-dimensional crystalline covalent triazine frameworks (2D-CTFs) using dual modulators.^23^ Dual modulators, aniline and a cosolvent, were used for dynamic covalent linkage formation and a noncovalent self-assembly process, respectively. This method is more effective to synthesize crystalline CTFs than *in situ* oxidation of alcohols with amidine–aldehyde polycondensation.^63^ Moreover, the crystalline 2D-CTFs possess high conversion and selectivity in the photocatalytic oxidation of aromatic sulfides into sulfoxides. Jin et al. also synthesized CTFs using benzyl halide and amidine monomers,^65^ and these CTFs have been successfully applied for efficient photocatalytic reforming of glucose for the first time, with a high hydrogen evolution rate up to 330 μmol g^–1^ h^–1^ under pH 12 (CTF-Br-2). Compared with monomers like benzyl alcohol, benzyl amine, and aldehyde used in the previous amidine–aldehyde condensation method, benzyl halides have higher availability and are more cost-effective. This work presents a new way to synthesize CTFs, which are promising materials for photocatalytic biomass reforming.

### 2.2 Synthesis of CTFs by Directly Introducing Triazine-Containing Monomers

Monomers containing triazine units as building blocks can also be used to synthesize CTFs. This strategy circumvents the harsh reaction conditions such as the application of high temperature, superacid, and strong bases to form triazine groups, and yields the modular nature of CTFs.

**Figure 10:**
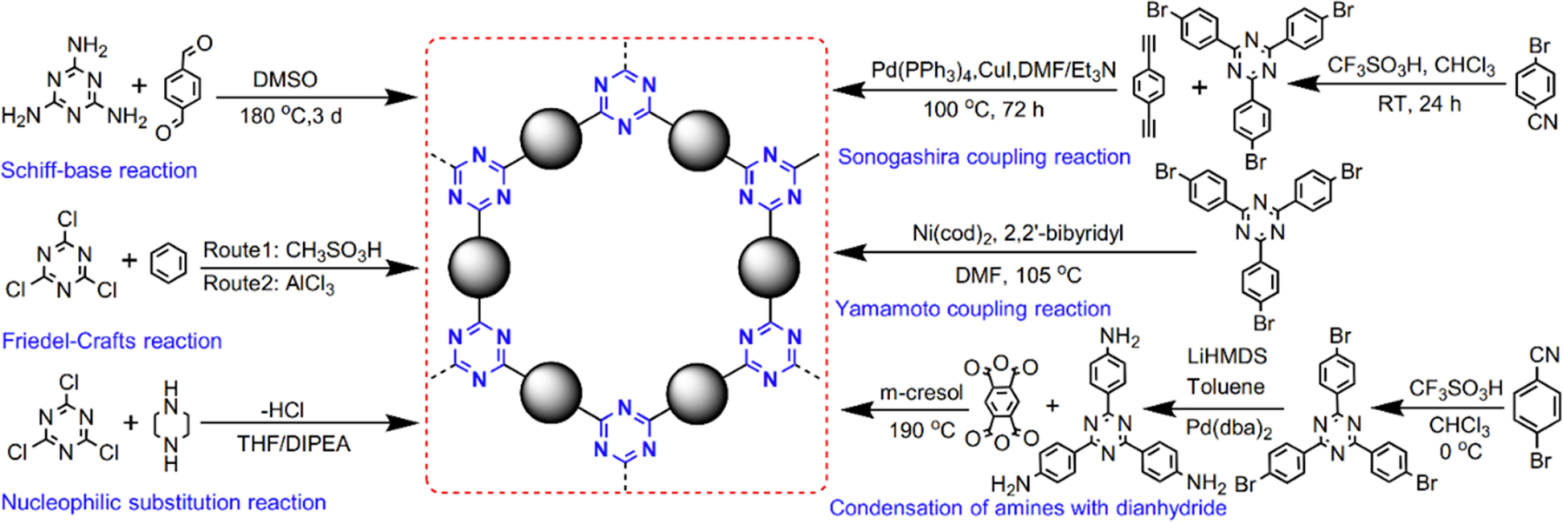
Typical synthesis of CTFs through the direct introduction of triazine units.

Various reactions have been developed for the direct introduction of triazine units (Figure 10). These reactions include a Schiff base reaction between melamine and aldehyde groups, nucleophilic substitution between melamine and nucleophilic reagents, a Friedel–Crafts alkylation reaction between melamine and aromatic rings, Sonogashira cross-coupling between bromine and alkyne groups, a Ni-catalyzed Yamamoto coupling reaction of aromatic bromine, and condensation reaction of amines and dianhydrides.

#### 2.2.1 Schiff Base Reaction

Schiff bases are a class of relatively stable imines with an alkyl or aryl group attached to the N atom with a formula of H_2_C=N—R (R is aryl or alkyl).^66^ Aromatic Schiff bases are usually obtained by nucleophilic addition reactions between aromatic amines and carbonyl compounds (e.g., aldehydes and ketones), forming hemiamine aldehydes similar to hemiacetals, followed by dehydration to form imines.

**Figure 11:**
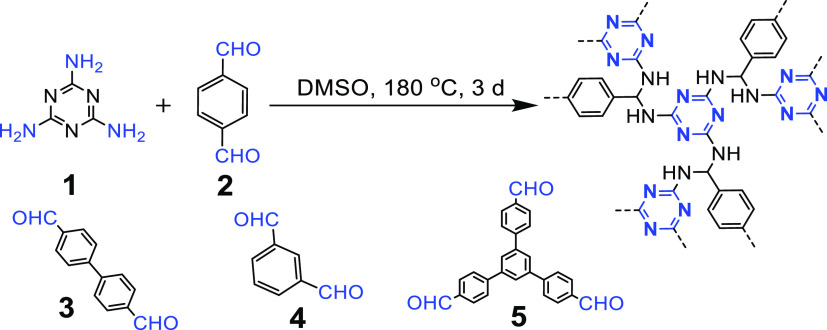
Synthesis of triazine-based polymer networks by Schiff base reaction. Adapted from ref (68). Copyright 2009 American Chemical Society.

The dynamic nature of the imine bonds in the Schiff base reaction enables the construction of complex molecular structures, and thus it can be used to “fix” triazine units for synthesizing CTFs.^67^ In 2009, Müllen et al. synthesized triazine-based polymer networks (Schiff base networks, SNWs) with amino-linked triazine units by the Schiff base reaction (Figure 11). This method uses the relatively inexpensive melamine as the starting material without using a catalyst.^68^ The obtained SNWs possess high surface areas and nitrogen contents higher than 40 wt %. Moreover, the SNWs show good stability under humid, acidic, and alkaline conditions and are stable up to 400 °C in a nitrogen atmosphere. Similarly, Bu et al. prepared a series of triazine-based porous organic polymers (aminal-linked porous organic polymers, APOPs) with acetal bonds by a simple condensation reaction between diaminotriazine and various benzaldehydes.^69^

**Figure 12:**
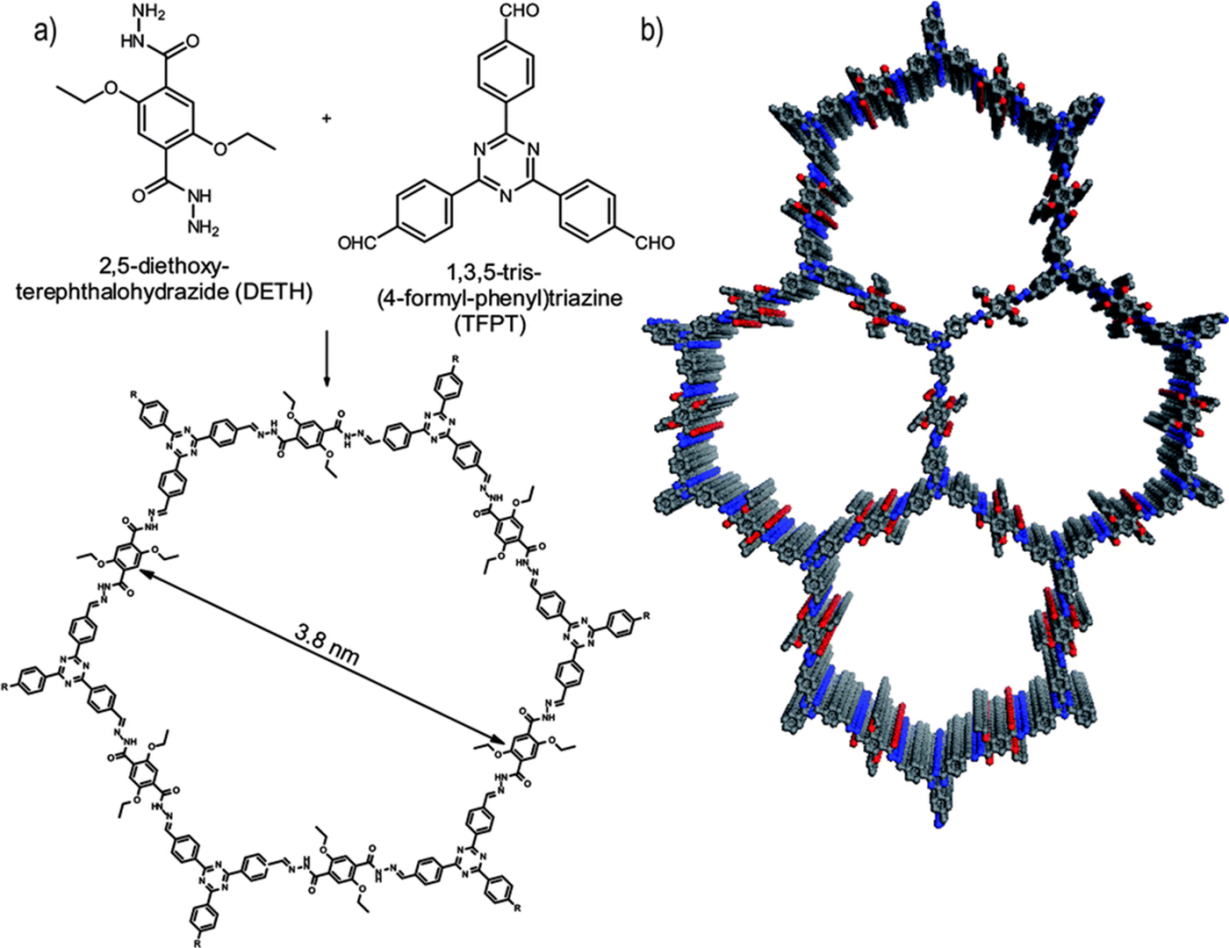
Acetic acid catalyzed hydrazone formation furnishes a mesoporous 2D network with a honeycomb type in-plane structure. (a) Scheme showing the condensation of the two monomers to form the TFPT-COF. (b) TFPT-COF with a cofacial orientation of the aromatic building blocks, constituting a close-to-eclipsed primitive hexagonal lattice (gray, carbon; blue, nitrogen; red, oxygen). Reprinted with permission from ref (71). Copyright 2014 The Royal Society of Chemistry.

Besides, a series of imine- or hydrazone-linked triazine-based porous organic materials can be synthesized by Schiff base reactions using aldehyde-containing triazine units with various amines. For example, Pitchumani et al. constructed imine-based triazine-based porous organic materials (mesoporous covalent imine polymerics, MCIPs) via the Schiff base reaction of 1,3,5-tris(4-formyl-phenyl)triazine (TFPT) and benzylamine/terephthalhydrazide.^70^ Subsequently, Lotsch et al. synthesized a COF containing hydrazone groups (TFPT-COF) using TFPT and 2,5-diethoxy-*p*-phenylene dihydrazide (DEFT) (Figure 12).^71^ The TFPT-COF has a hexagonal pore arrangement structure with a specific surface area of 1360 m^2^ g^–1^ and an average pore size of 3.8 nm. Meanwhile, the TFPT-COF exhibits good stability in solvents such as methanol and dichloromethane.

#### 2.2.2 Friedel–Crafts Reaction

In the Friedel–Crafts reaction, the H atoms on aromatic rings in aromatic hydrocarbons are replaced by alkyl groups or acyl groups with anhydrous AlCl_3_ as a catalyst, forming alkyl hydrocarbons.^73^ A series of hydrazone- or imine-linked triazine-based polymer networks can be synthesized by the Friedel–Crafts reaction using aldehyde-containing triazine units with various amines. Thus, CTFs can also be synthesized by a relatively simple Friedel–Crafts reaction between melamine and aromatic structural units.^74^

**Figure 13:**
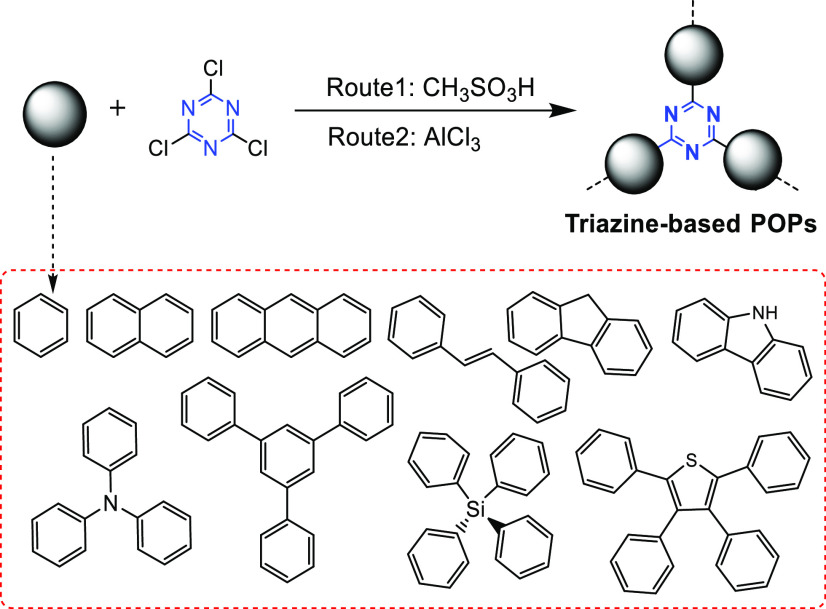
Synthesis of triazine-based porous materials via Friedel–Crafts reaction. Adapted with permission from ref (74). Copyright 2016 The Royal Society of Chemistry.

**Figure 14:**
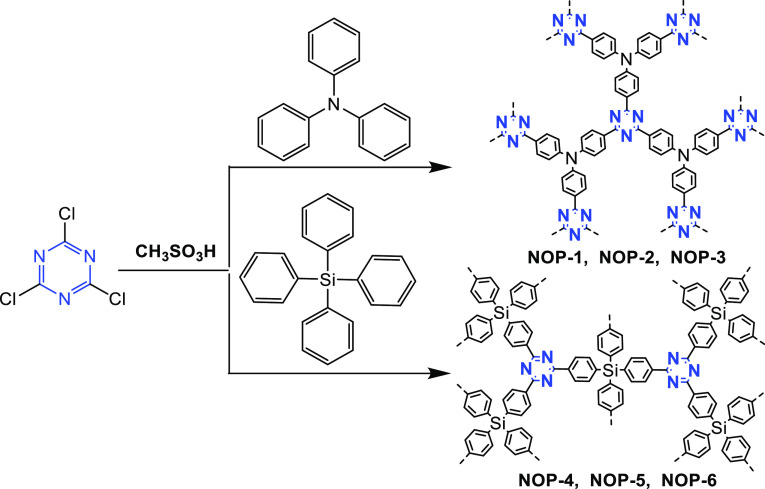
Synthetic routes of the NOP networks. Adapted with permission from ref (56). Copyright 2013 The Royal Society of Chemistry.

Either methanesulfonic acid (Figure 13, route 1) or anhydrous AlCl_3_ (Figure 13, route 2) is used as the catalyst for Friedel–Crafts reaction based polymerization. For example, methanesulfonic acid is used to catalyze the Friedel–Crafts reactions between melamine and triphenylamine or tetraphenylsilane, respectively, to synthesize novel triazine-based nanoporous organic polymers (nanoporous organic polymers, NOP-1–NOP-6) (Figure 14).^56^ As a result of the triazine units’ strong electron-accepting ability and high symmetry, the NOPs possess excellent photophysical properties and are promising fluorescent probes for the detection of heavy metals.^56^ Besides, AlCl_3_ is used to catalyze the polymerization of melamine with various aromatic compounds in dichloromethane solution (Figure 13, route 2).^75^

Compared to other synthesis methods, Friedel–Crafts reaction based polymerization is simple, convenient, and inexpensive. Since cyanuric chloride contains the triazine units, nitrile monomers are not necessary for CTFs synthesis by Friedel–Crafts alkylation of cyanuric chloride, and the possible monomers for CTFs synthesis is broadened. However, these classical Friedel–Crafts reactions often generate polysubstituted and rearranged byproducts, which greatly limits their scalability ecologically.

A mechanochemical Friedel–Crafts alkylation reaction has also been applied for CTFs synthesis.^55^ Mechanochemistry is an emerging discipline that studies chemical reactions and physicochemical properties or changes in the internal microstructures of materials induced by mechanical forces.^76^ Mechanochemical synthesis is fast with low energy consumption. Generally, mechanical forces are generated by grinding, extrusion, shearing, or friction to induce changes in the chemical and physical properties of reactants, chemically transforming substances into products.^77^

**Figure 15:**
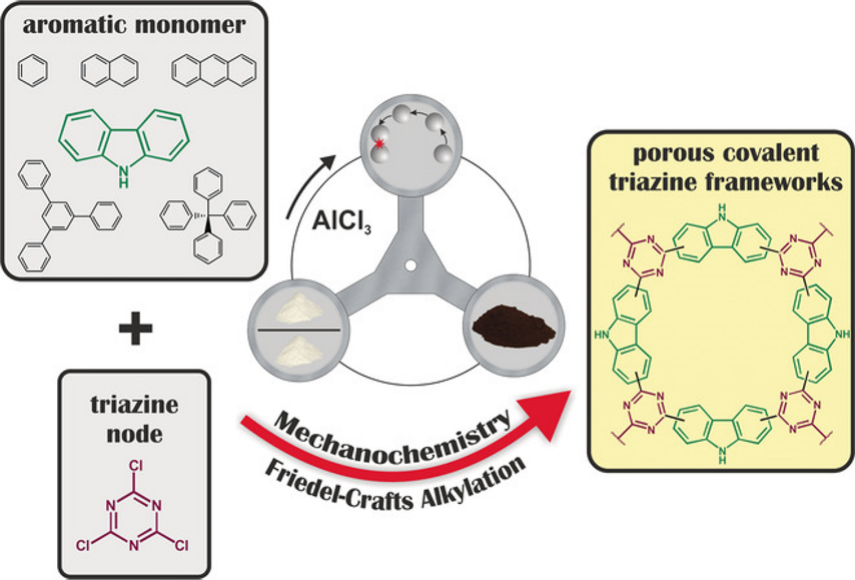
Mechanochemical synthesis of CTFs by Friedel–Crafts reaction between different aromatic monomers and melamine. Adapted with permission from ref (78). Copyright 2017 John Wiley and Sons.

Recently, Borchardt et al. synthesized CTFs using a mechanochemical Friedel–Crafts alkylation reaction (Figure 15).^78^ Melamine as the triazine node, electron-rich aromatic compounds as nucleophilic reagents, AlCl_3_ as an activator, and ZnCl_2_ as a filler were applied. A series of CTFs were synthesized in less than 3 h with ultrahigh yields. This method provides a new route for the bulk synthesis of new CTFs. Moreover, mechanochemical synthesis is a solvent-free, efficient, and easily scalable synthesis method and is expected to replace the traditional solvent thermal synthesis routes. However, the substitution selectivity of the electron-rich aromatic monomer sites is not unique through the Friedel–Crafts alkylation reaction, and therefore the structure of the material cannot be precisely controlled leading to disordered structures.

#### 2.2.3 Nucleophilic Substitution Reactions

Nucleophilic substitution reaction refers to a reaction in which a negatively or weakly negatively charged nucleophile attacks (or strikes) and replaces a positively or partially positively charged carbon nucleus on a target molecule.^79,80^ If the positively charged carbon core is replaced by a nucleophile already bearing triazine units, CTFs can be obtained.

**Figure 16:**
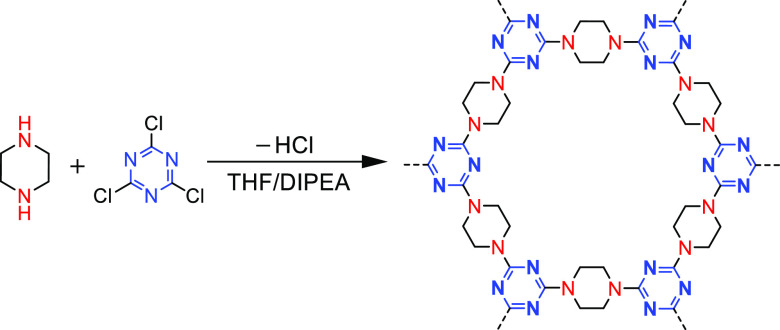
Scheme of PAF-6 synthesis. Adapted with permission from ref (81). Copyright 2011 The Royal Society of Chemistry.

**Figure 17:**
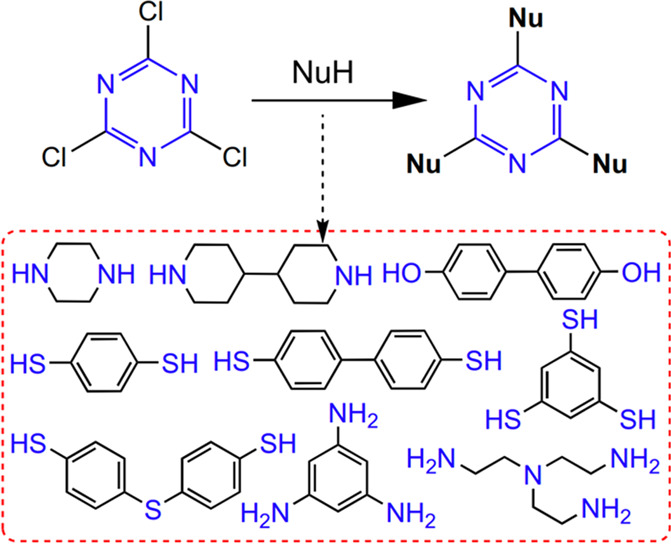
Scheme of the synthesis of triazine-based porous organic materials via nucleophilic substitution reaction. Adapted with permission from ref (74). Copyright 2016 The Royal Society of Chemistry.

Trichlorocyanine is a versatile and relatively inexpensive chemical. Due to its planar, rigid, and high *D*_3*h*_ symmetry structure, different nucleophilic reagents can chemically and selectively substitute the Cl atoms in its structure. Thus, trichlorocyanine can be used as a source for the synthesis of triazine rings in CTFs. In 2011, Zhu et al. constructed two-dimensional CTFs (porous aromatic frameworks, PAF-6) with melamine as a planar triangular building block, by nucleophilic substitution using piperazine as a linear linker molecule (Figure 16).^81^ A variety of triazine-based porous organic polymers using nucleophilic substitution are also developed by changing the structure of the linker molecules (Figure 17).^74^ This method can be applied under mild reaction conditions without using a metal catalyst, providing a cheap and simple way for the synthesis of new porous organic polymer materials.

#### 2.2.4 Coupling Reactions

##### 2.2.4.1 Sonogashira Coupling Reaction

**Figure 18:**
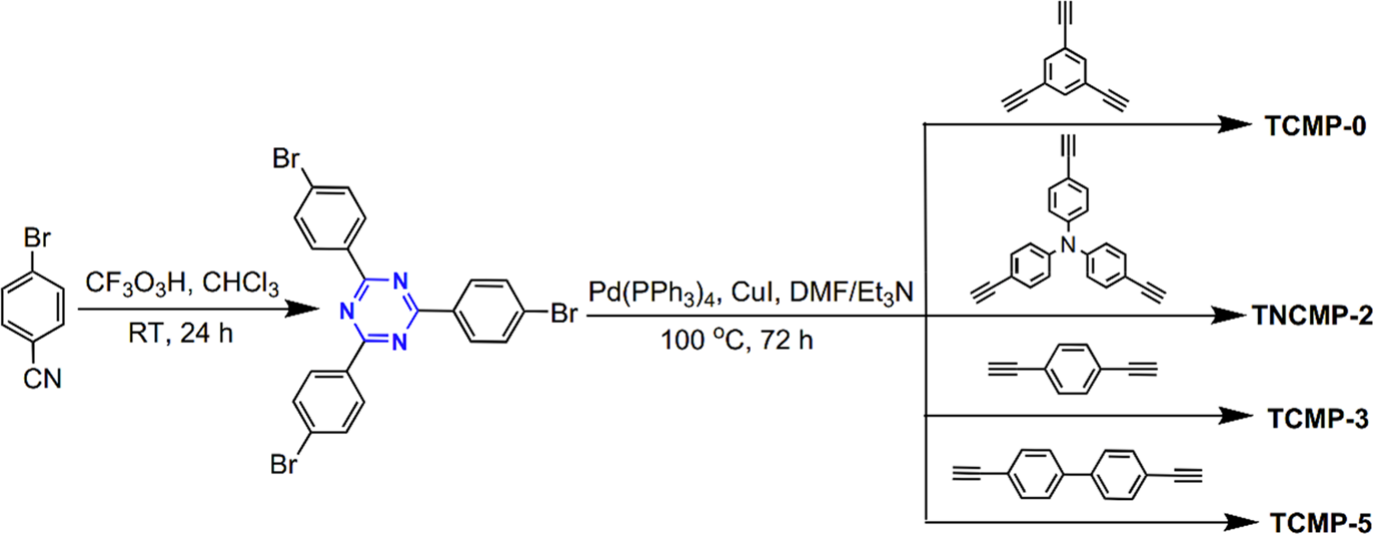
Triazine monomer and synthetic route of TCMPs via Sonogashira coupling reaction. Adapted with permission from ref (20). Copyright 2011 The Royal Society of Chemistry.

The Sonogashira coupling reaction is a cross-coupling reaction used in organic synthesis to form C–C bonds.^82^ A Pd catalyst, as well as a Cu cocatalyst, is used to catalyze the formation of a C–C bond between a terminal alkyne and an aryl or vinyl halide.^82^ Cooper et al. synthesized conjugated microporous polymers based on electron-absorbing 1,3,5-triazine nodes via a Pd-catalyzed Sonogashira–Hagihara cross-coupling reaction.^20^ With the use of 2,4,6-tris(4-bromophenyl)-1,3,5-triazine as a monomer with various di/triacetylenes, brown CTF powders with yields higher than 90% were obtained (Figure 18). The obtained amorphous 1,3,5-triazine node conjugated microporous polymers (TCMPs) exhibited very high thermal and chemical stabilities under aqueous conditions. Besides, the specific surface areas of the TCMPs reach 494–995 m^2^ g^–1^. Moreover, the polymeric reticulation networks TNCMP-2 and TCMP-3 possess highly microporous structures, while the TCMP-0 and TCMP-5 networks were mesoporous. Notably, TNCMP-2 is a polymer composed of an electron acceptor (i.e., 1,3,5-triazine) and an electron donor (i.e., triphenylamine), and therefore it may have optoelectronic properties.

##### 2.2.4.2 Yamamoto Coupling Reaction

Yamamoto coupling or Yamamoto polymerization refers to coupling polycondensation or dehalogenative C–C coupling reactions between dihalogenated aromatic hydrocarbons and polyhalogenated aromatic hydrocarbons through dehalogenation reactions.^83^ Transition metal reagents such as NiCl_2_(bipy) and Ni(cod)_2_ are commonly used as catalysts to obtain polyaromatic polymers and cyclic oligomerization products.^84^ If the halogenated hydrocarbons contain triazine units, the Yamamoto coupling reaction can be used to synthesize CTFs.

**Figure 19:**
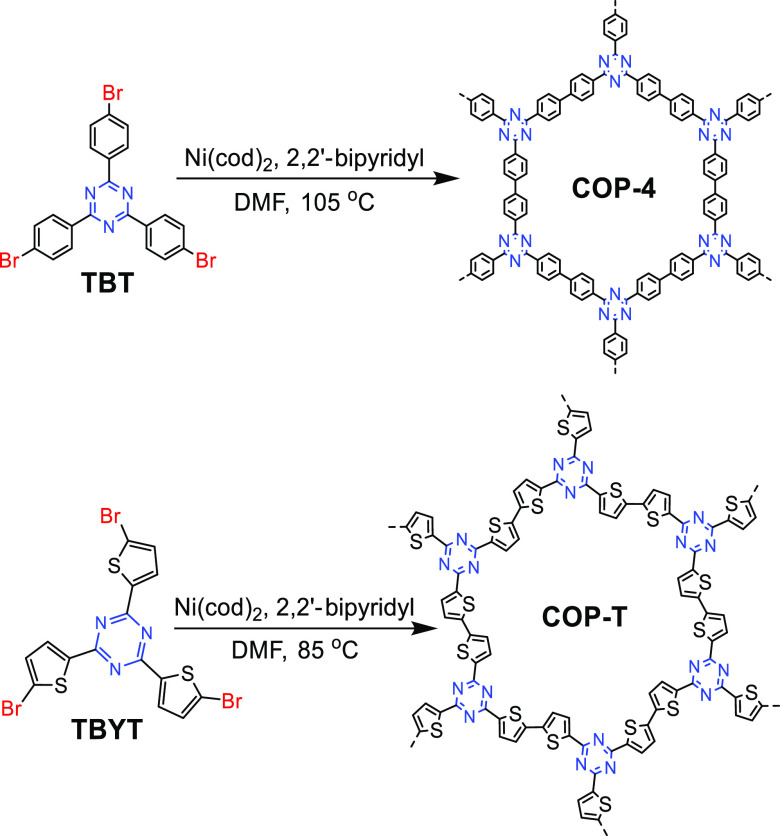
Schematic diagram of the synthesis of COP-4 and COP-T via Yamamoto coupling reaction. Adapted with permission from ref (86). Copyright 2014 John Wiley and Sons.

In 2012, Cao et al. synthesized a porous luminescent CTF, COP-4 (covalent organic polymer, COP), via self-condensation of 2,4,6-tris(4-bromo-phenyl)-1,3,5-triazine monomer by a Ni-catalyzed Yamamoto coupling reaction.^85^ Subsequently, they also synthesized COP-T (T = 2,4,6-tris(5-bromothiophen-2-yl)-1,3,5-triazine) by the self-coupling of 2,4,6-tris(5-bromothiophen-2-yl)-1,3,5-triazine (Figure 19).^86^ Both porous CTFs are connected by stable covalent C–C bonds and possess high hydrothermal stability as well as graphene-like layered structures.

##### 2.2.4.3 Suzuki Coupling Reaction

The Suzuki–Miyaura reaction or Suzuki coupling reaction is a cross-coupling reaction and has been widely used to synthesize polyolefins, styrenes, and substituted biphenyls.^87,88^ In this reaction, a C(sp^2^)–C(sp) single bond is formed by coupling a halide (R^1^–X) with an organoboron species (R^2^–BY_2_) using a Pd catalyst and a base for promoting metal transfer.^89^

**Figure 20:**
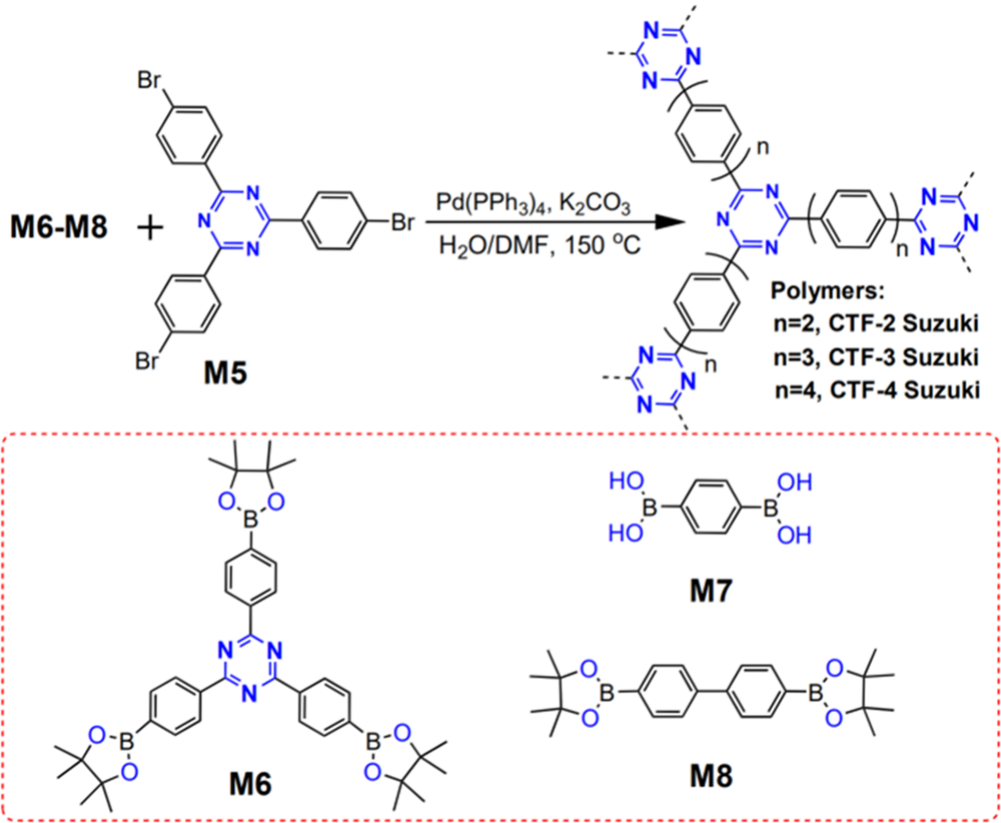
Synthetic scheme for covalent triazine-based frameworks via Suzuki coupling reaction. Adapted with permission from ref (90). Copyright 2017 Elsevier.

In 2017, Cooper et al. synthesized a series of CTFs via Pd(0)-catalyzed Suzuki–Miyaura polycondensation of 2,4,6-tris(4-bromophenyl)-1,3,5-triazine (M5) and 2,4,6-tris-[4-(4,4,5,5-tetramethyl-1,3,2-dioxaborolan-2-yl)phenyl]-1,3,5-triazine (M6), 1,4-benzene diboronic acid (M7), or 4,4′-biphenyldiboronic acid bis(pinacol) ester (M8) in *N*,*N*-dimethylformamide at 150 °C in the presence of aqueous K_2_CO_3_, obtaining CTF-2–CTF-4 Suzuki polymer powders (Figure 20).^90^ After ball milling, the average particle size of the CTF-2 Suzuki and CTF-3 Suzuki polymer powders decreases from several millimeters to a few hundred micrometers, while that of the CTF-4 Suzuki polymer powder remains unchanged, probably due to its softness and lower network density. UV–visible spectra demonstrate that the band gap of the obtained CTFs depends on the length of the 1,4-phenylene linker between the triazine cores. The longer the 1,4-phenylene linker, the narrower CTFs’ band gap. From the CTF-2 Suzuki polymer powder to the CTF-4 Suzuki polymer powder, the band gap decreases from 2.93 to 2.85 eV. Under visible-light irradiation (>420 nm), CTF-2 Suzuki and CTF-3 Suzuki polymer powders are active for hydrogen evolution reaction.^90^

##### 2.2.4.4 Knoevenagel Reaction

The Knoevenagel reaction belongs to the general class of base-catalyzed aldol-type condensations, in which a carbanion adds to a carbonyl or heterocarbonyl group. Generally, aldehydes and ketones react with active methylene compounds in the presence of a weak base such as an amine, producing alkylidene/benzylidene dicarbonyls or analogous compounds. With the use of a substrate containing a triazine unit, CTFs polymers can be obtained by the Knoevenagel reaction.^91^

**Figure 21:**
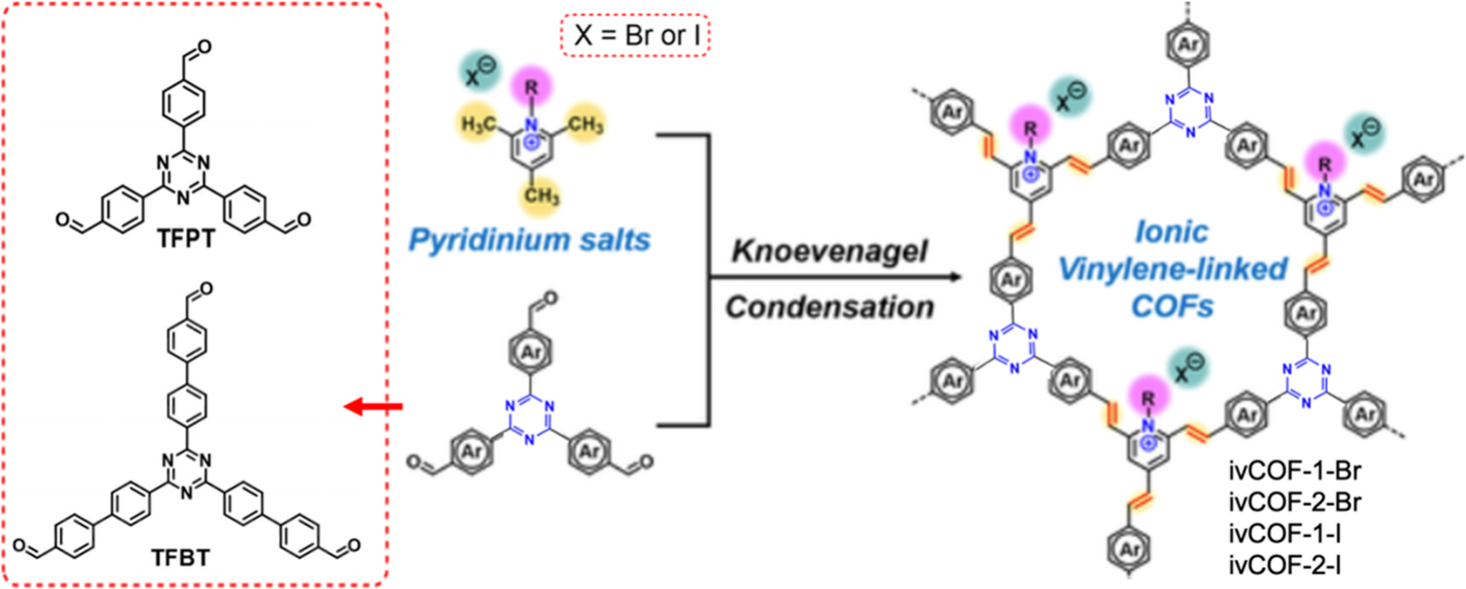
Synthesis of ivCOF-X via Knoevenagel reaction. ivCOF-1-Br and ivCOF-2-Br indicate COFs prepared by ETMP-Br with TFPT and TFBT, respectively. ivCOF-1-I and ivCOF-2-I indicate COFs prepared by ETMP-I with TFPT and TFBT, respectively. Adapted with permission from ref (92). Copyright 2021 John Wiley and Sons.

In 2021, Zhang et al. reported a new family of ionic vinylene linked two-dimensional (2D) COFs synthesized through the Knoevenagel condensation of *N*-ethyl-2,4,6-trimethylpyridinium bromide (ETMP-Br) or iodide (ETMP-I) with tritopic aromatic aldehyde derivatives 1,3,5-tris(4-formylphenyl)triazine (TFPT) and 1,3,5-tris(4′-formyl-biphenyl-4-yl)triazine (TFBT) (Figure 21).^92^ The resulting COFs possess honeycomb-like 2D structures and large surface areas (as large as 1343 m^2^ g^–1^) with regular open channels (1.4 and 1.9 nm diameters). By virtue of their well-defined ionic frameworks, the as-synthesized COFs can be uniformly composited with poly(ethylene oxide) (PEO) and lithium bis(trifluoromethyl sulfonyl)imide (LiTFSI), displaying satisfactory lithium ion (Li ion) conductivity potentially applicable to a wide range of tasks, such as energy storage and environmental protection.

##### 2.2.4.5 Tröger’s Base Formation Reaction

Tröger’s base (TB) refers to 2,8-dimethyl-6*H*,12*H*-5,11-methanodibenzo[*b*,*f*][1,5]diazocine. TB is known as the bridged bicyclic diamine between 4-methylaniline (*p*-toluidine) and formaldehyde in the acid-mediated analogous reaction between aniline and formaldehyde, first reported by Julius Tröger in 1887.^93−95^ Despite its long history, the TB-forming reaction has only recently been used to synthesize polymers.^96,97^

**Figure 22:**
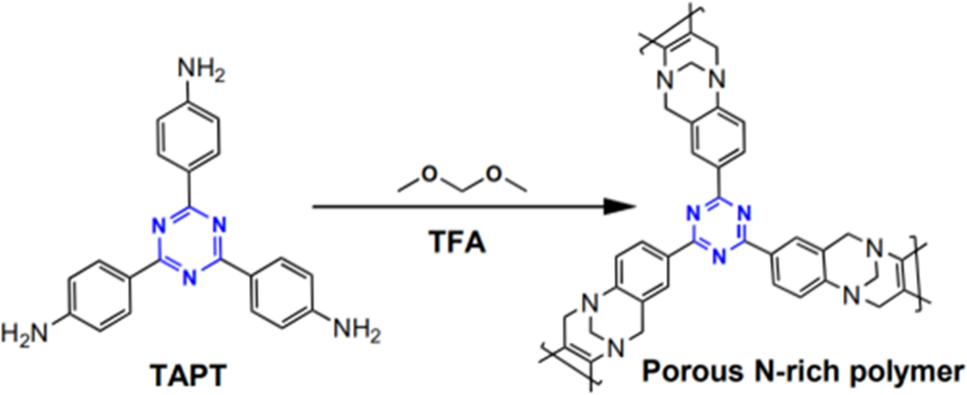
Synthesis of porous N-rich polymer from 2,4,6-tris(4-aminophenyl)-*s*-triazine via Tröger’s base formation reaction. Adapted with permission from ref (98). Copyright 2018 The Royal Society of Chemistry.

In 2018, Yu et al. synthesized the porous organic polymers containing Tröger’s base and *s*-triazine group building blocks through the reaction of 2,4,6-tris(4-aminophenyl)-*s*-triazine (TAPT) and dimethoxymethane in trifluoroacetic acid (TFA) solution at room temperature (Figure 22).^98^ This polymer exhibits porous properties with a BET surface area of 473.1 m^2^ g^–1^ and a high CO_2_ adsorption capacity (CO_2_ uptake of 49.8 cm^3^ g^–1^ at 1 bar and 273 K) at ambient pressure. Due to the presence of Tröger’s base and the *s*-triazine group, it shows high isosteric heat (i.e., 33.7 kJ mol^–1^) for CO_2_, leading to a higher CO_2_ adsorption selectivity over N_2_ and CH_4_. Moreover, this polymer exhibits colorimetric detection performance for naked-eye detection of HCl gas.^98^

##### 2.2.4.6 Stille Cross-Coupling Reaction

**Figure 23:**
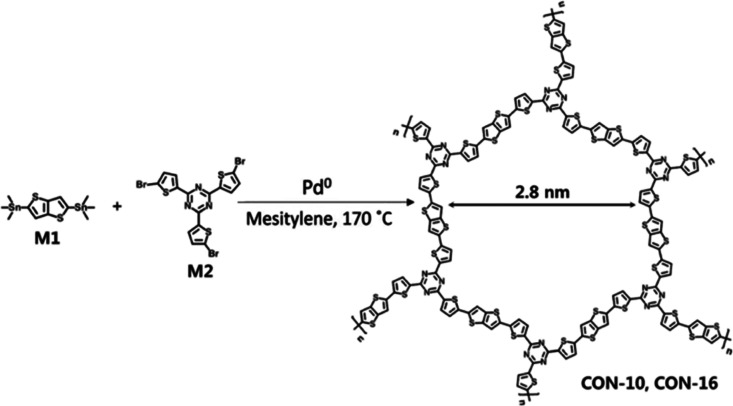
Schematic representation for the solvothermal synthesis of CON-10 and CON-16 via a Stille cross-coupling reaction. Adapted with permission from ref (99). Copyright 2017 The Royal Society of Chemistry. Adapted with permission from ref (100). Copyright 2022 The Royal Society of Chemistry.

In 2017, Kim et al. synthesized two kinds of triazine-based covalent organic polymers (i.e., CON-10 and CON-16) via two different Stille cross-coupling reaction routes using 2,5-bis(trimethylstannyl)thieno-(3,2-*b*)thiophene (M1) and 2,4,6-tris(5-bromothiophen-2-yl)-1,3,5-triazine (M2) as monomers (Figure 23).^99^ CON-10 was synthesized using Pd metal as a catalyst in mesitylene as the solvent and was refluxed for 3 days, while CON-16 was obtained with the same substrates, but the reaction was performed in an ampule in a glovebox The obtained CONs possess mesopores with a diameter of approximately 2.8 nm and a band gap of 1.91 eV, and CON-16 possesses a relatively higher surface area compared to CON-10.^99,100^

#### 2.2.5 Amine–Dianhydride Condensation

**Figure 24:**
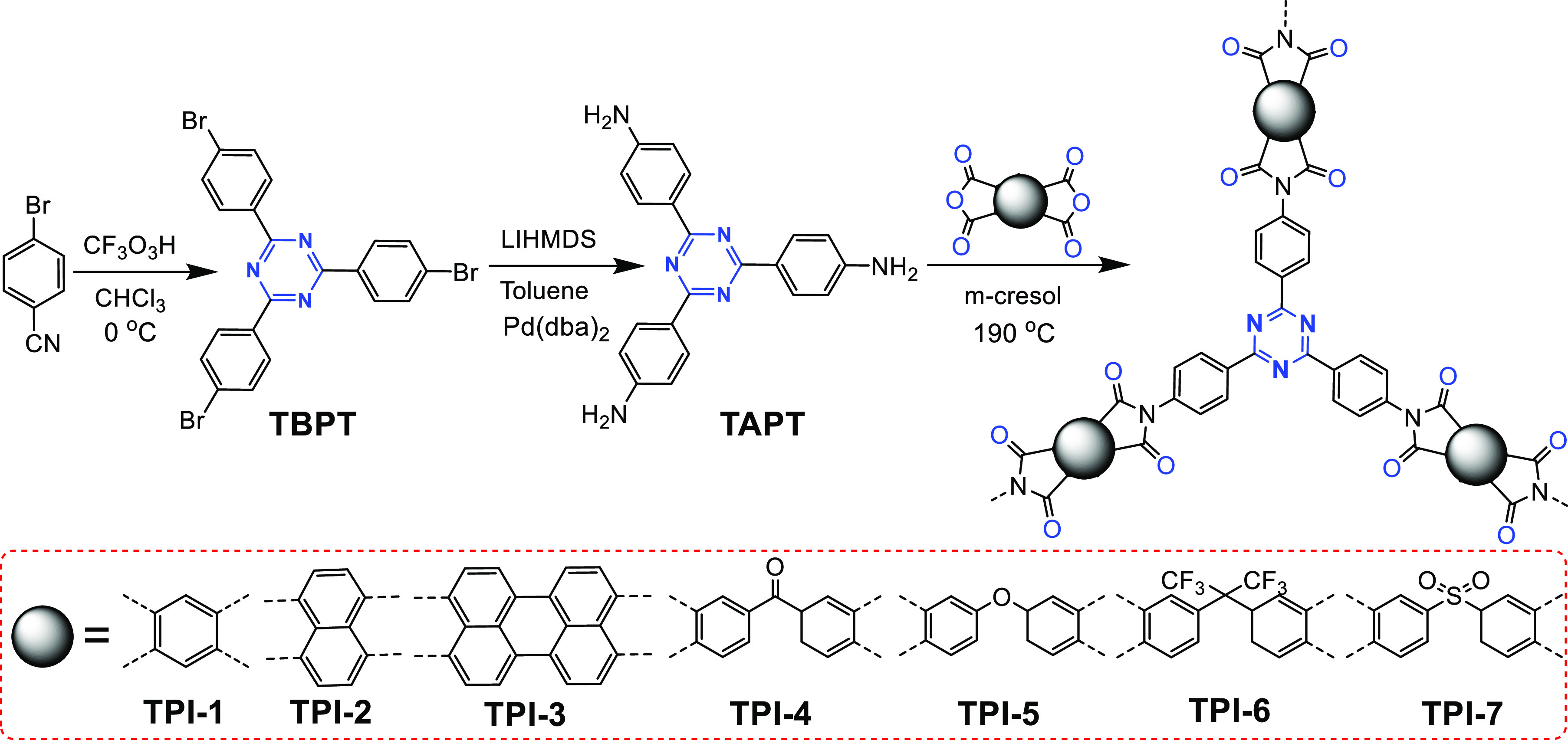
Synthetic route of TPIs via amine–dianhydride condensation. Adapted from ref (13). Copyright 2013 American Chemical Society.

In 2013, Senker et al. synthesized a network of seven types of microporous triazinyl polyimides (triazinyl polyimides, TPIs) by the condensation of 2,4,6-tris(*p*-aminophenyl)-1,3,5-triazine (TAPT) with various dianhydrides (Figure 24).^13^ The obtained TPI polymers exhibited high thermal and chemical stabilities in air. Ar physisorption results showed that TPI-1 and TPI-2 have large specific surface areas of 809 and 796 m^2^ g^–1^, respectively, but TPI-4, TPI-5, and TPI-6 have relatively low specific surface areas. This is because the single bonds connecting the molecular centers increase the flexibility of the network. The resulting less rigid framework causes partial collapse of the pores, thus reducing the accessible surface area of the polymers.

This synthetic strategy allows the construction of various polyimide networks by simply changing the type of monomer. In 2017, Park et al. synthesized hyperbranched polyimide networks by condensation of tetra(4,6-diamino-*s*-triazine-2-yl)tetraphenyl methane (M1) with 1,4,5,8-naphthalenetetracarboxylic anhydride (NTCDA) in DMSO solution.^101^ The diaminotriazine fraction in M1 can provide an effective branched site, giving the synthesized CTFs a specific surface area of 1150 m^2^ g^–1^. It is worth noting that the void structure of the polyimide porous organic polymers synthesized by Park et al. can be modified by supercritical CO_2_ surface activation to tune the pore structure.

## 3 Conclusions and Future Outlook

CTFs are promising materials for applications in gas adsorption and separation, heterogeneous catalysis, photocatalysis, and supercapacitors due to the simple synthesis method, and designable structure, as well as adjustable specific surface area and pore structure. Until now, CFTs with different colors (i.e., black, yellow, green, white, red, etc.) and specific areas of 2–2475 cm^2^ g^–1^ have been prepared by various methods, including ionothermal polymerization, superacid catalyzed polymerization, aromatic amide condensation, hard-template-assisted synthesis, amidine–aldehyde condensation, Schiff base reaction, Friedel–Crafts reaction, nucleophilic substitution reaction, Sonogashira coupling reaction, Yamamoto coupling reaction, and amine–dianhydride condensation (Table S1). Even though great advances have been made in the synthesis of CTFs, there is still room for further improvement to push CTFs synthesis to large-scale production and widen the application of CTFs in more fields.

### 3.1 Avoiding Carbonization

CTFs are formed through the linkage of strong triazine bonds and are generally synthesized by ionothermal polymerization. Most of the obtained CTFs exhibit remarkable stability and porosity, but the harsh synthesis conditions, especially the high temperature, lead to carbonization, influencing their optical properties and limiting their utilization. Currently, the carbonization of CTFs cannot be eliminated by the ionothermal synthesis method and aromatic amide polymerization method. The carbonization degree of the material can be decreased by lowering the synthesis temperature. The amidine–aldehyde condensation and superacid polymerization can overcome the effect of carbonization.

### 3.2 Increasing Crystallinity

CTFs synthesized by most methods are amorphous, but the crystallinity has a great impact on the optical properties of CTFs. For example, crystalline CTFs generally perform better than amorphous CTFs in light absorption and photocatalysis. Superacid polymerization and *in situ* oxidation of alcohols with amidine condensation led to crystalline CTFs. However, there are only a few successful examples. Therefore, inspired by the condensation of *in situ* oxidative alcohols with amidines, more strategies to synthesize CTFs with higher crystallinity under mild reaction conditions and open systems remain to be explored.

### 3.3 Doping with Heteroatoms

Functional groups containing specific functions can be introduced in the synthesis to increase the surface alkalinity, adjust the pore size, and incorporate dopants. For example, the introduction of N-containing functional groups (e.g., amines, amides, pyridines, and imidazoles) improves the interaction between the metal and the carrier and enables immobilization of nanoparticles and molecular active species, increasing the number of active sites, thus enhancing the catalytic activity or the absorption of gases. In addition, S dopant (e.g., thiophene) can enhance CTFs’ performance in the absorption range and intensity of light.

### 3.4 Becoming Safer

Cyano monomers used for CTFs synthesis are generally toxic. During ionothermal polymerization, there is also a risk of ampule explosion in the synthesis by the generation of escaping gases. In superacid polymerization, TFMS as one of the strongest acids is strongly corrosive and toxic. In recent years, the synthesis of CTFs by *in situ* oxidation of aldehyde groups as well as alcohol groups with amidine condensation has been carried out under mild synthesis conditions and is relatively safe. Therefore, developing safer methods is the trend of CTFs material research and development.

### 3.5 Versatile Future Applications

As a new class of covalent organic frameworks, CTFs, possessing excellent chemical and structural properties, are promising for a wide range of applications such as organic synthesis, gas adsorption, catalysis, and energy materials. First, the porous structure and large specific surface area enable the application of CTFs in the adsorption of organic dyes and the separation of mixed gases. Second, because of the low skeleton density, high specific surface area, and microporous pore structure, CTFs are considered promising hydrogen storage materials. Third, due to the strong covalent bonds (i.e., C=N) in the structure, CTFs possessing high thermal and chemical stabilities are ideal materials as catalyst supports for liquid-phase reactions. Fourth, owing to the N content, porous structure, large specific surface area, and low resistivity, CTFs have been developed as capacitive electrode materials for supercapacitors. Fifth, the tailored optical and semiconductive properties of CTFs make them promising metal-free polymeric photocatalysts.

In conclusion, CTFs are promising covalent organic polymers with properties such as being nitrogen-rich, highly stable, and porous due to their special triazine structural units and are expected to be some of the most promising porous materials for industrial applications with the continuous development and improvement of various synthetic methods.

## Supporting Information Available

Comparison of typical CTFs synthesis methods (PDF)
