# Supplementary material for: Advances in the Synthesis of Covalent Triazine Frameworks
Source: ACS Omega. 2023 Jan 23;8(5):4527–42. doi: 10.1021/acsomega.2c06961 (PMC9909813; doi:10.1021/acsomega.2c06961)
Supplement: Supplementary file 1 — ao2c06961_si_001.pdf [file ao2c06961_si_001.pdf]

Advances in the Synthesis of Covalent Triazine Frameworks

Longfei Liao[1,2*], Mingyu Li[1], Yongli Yin[2], Jian Chen[3], Qitong Zhong[3], Ruixing Du[3], Shuilian Liu[3], Yiming He[3], Weijie Fu[3], Feng Zeng[3*]

[1] School of Materials Science and Engineering, Harbin Institute of Technology (Shenzhen), Shenzhen 518055, Guangdong, China.

[2] Space Science and Technology Institute (Shenzhen), Shenzhen 518117, Guangdong, China.

[3] State Key Laboratory of Materials-Oriented Chemical Engineering, College of Chemical Engineering, Nanjing Tech University, Nanjing 211816, Jiangsu, China.

[*] Corresponding authors.

E-mail address: liaolongfei123@126.com (Longfei Liao); zeng@njtech.edu.cn (Feng Zeng)

Table S1. A comparison of the typical CTFs synthesis methods.

Methods	Typical CTFs	Colour	S_{BET} [cm^2 g^{-1}]	Ref.
Ionothermal polymerization	CTF-1-1.0	Black	791	1
	CTF-1-0.1	Black	1123	1
	CTF-DCBP-0.1	Black	2475	1
	CTF-DCT-0.1	Black	584	1
	CTF-DCP-0.1	Black	730	1
	CTF-TCT-0.1	Black	975	1
Super acid polymerization	P1	Light yellow	2	2
	P2	Light green	776	2
	P3	Llight pale	571	2
	P4	Off-white	867	2
	P5	Yellow	960	2
	P6	Yellow	1152	2
	P1M	Pale yellow	4	2
	P2M	Yellow	464	2
	P3M	Pale yellow	523	2
	P4M	Pale orange	542	2
	P5M	Grey/black	542	2
	P6M	Light yellow	947	2
Aromatic amide condensation	*p*CTF-1	Black	2034	3
Hard template-assisted synthesis	Hollow CTF-B	N.A.	565	4
	Bulk CTF-B	N.A.	687	4
	Hollow CTF-BT	N.A.	90	4
	Bulk CTF-BT	N.A.	67	4

	CTF-Th@SBA-15	Yellow	548	[5]
	CTF-Th	N.A.	57	[5]
Amidine-aldehyde condensation	CTF-HUST-1	Yellow	663	[6]
	CTF-HUST-2	Yellowish	757	[6]
	CTF-HUST-3	Orange	807	[6]
	CTF-HUST-4	Light orange	764	[6]
	CTF-HUST-C1	Yellow	599	[7]
	CTF-HUST-C1-100	Yellow	151	[7]
	CTF-HUST-C1-120	Yellow	467	[7]
	CTF-HUST-C1-150	Yellow	467	[7]
	CTF-HUST-C1-180	N.A.	-	[7]
	CTF-HUST-C5	Yellow	520	[7]
	CTF-HUST-C6	Yellow	598	[7]
Schiff base reaction	SNW-1	Off-white	1377	[8]
	SNW-2	N.A.	842	[8]
	SNW-3	N.A.	1133	[8]
	SNW-4	N.A.	1213	[8]
	APOP-1	N.A.	1298	[9]
	APOP-2	N.A.	906	[9]
	APOP-3	N.A.	1402	[10]
	APOP-4	N.A.	833	[11]
Friedel-Crafts reaction	NOP-1	Yellow-brown	749	[12]
	NOP-2	Yellow-brown	803	[12]
	NOP-3	Yellow-brown	894	[12]
	NOP-4	Khaki	428	[12]
	NOP-5	Khaki	613	[12]
	NOP-6	Khaki	720	[12]
Nucleophilic substitution reactions	PAF-6	N. A	182.7	[13]
Sonogashira coupling reaction	TCMP-0	Brown	963	[14]
	TCMP-2	Dark-brown	995	[14]
	TCMP-3	Brown	691	[14]
	TCMP-5	Brown	494	[14]
	COP-2	Cream	1986	[15]

Yamamoto coupling reaction	COP-3	Cream	1869	[15]
	COP-4	Cream	2015	[15]
	COP-T	N.A.	N.A.	[16]
	COP-P	N.A.	622	[16]
Amine-dianhydride condensation	TPI-1	Yellowish	809	[17]
	TPI-2	Brown	796	[17]
	TPI-3	Dark red	40	[17]
	TPI-4	N.A.	245	[17]
	TPI-5	N.A.	201	[17]
	TPI-6	N.A.	510	[17]
	TPI-7	N.A.	<10	[17]

Reference

(1) Pierre Kuhn; Markus Antonietti; Arne Thomas. Porous, Covalent Triazine-Based Frameworks Prepared by Ionothermal Synthesis. *Angew. Chem. Int. Ed.* **2008**, *47* (18), 3450–3453. https://doi.org/10.1002/anie.200705710.

(2) Shijie Ren; Michael J. Bojdys; Robert Dawson; Andrea Laybourn; Yaroslav Z. Khimyak; Dave J. Adams; Andrew I. Cooper. Porous, Fluorescent, Covalent Triazine-Based Frameworks Via Room-Temperature and Microwave-Assisted Synthesis. *Adv. Mater.* **2012**, *24* (17), 2357–2361. https://doi.org/10.1002/adma.201200751.

(3) Yu, S.-Y.; Mahmood, J.; Noh, H.-J.; Seo, J.-M.; Jung, S.-M.; Shin, S.-H.; Im, Y.-K.; Jeon, I.-Y.; Baek, J.-B. Direct Synthesis of a Covalent Triazine-Based Framework from Aromatic Amides. *Angew. Chem. Int. Ed.* **2018**, *57* (28), 8438–8442. https://doi.org/10.1002/anie.201801128.

(4) Wei Huang; Zi Jun Wang; Beatriz Chiyin Ma; Saman Ghasimi; Dominik Gehrig; Frédéric Laquai; Katharina Landfester; Kai A. I. Zhang. Hollow Nanoporous Covalent Triazine Frameworks via Acid Vapor-Assisted Solid Phase Synthesis for Enhanced Visible Light Photoactivity. *J. Mater. Chem.* **2016**, *4* (20), 7555–7559. https://doi.org/10.1039/C6TA01828A.

(5) Wei Huang; Beatriz Chiyin Ma; Hao Lu; Run Li; Lei Wang; Katharina Landfester; Kai A. I. Zhang. Visible-Light-Promoted Selective Oxidation of Alcohols Using a Covalent Triazine Framework. *ACS Catal.* **2017**, *7* (8), 5438–5442. https://doi.org/10.1021/acscatal.7b01719.

(6) Kewei Wang; Li-Ming Yang; Xi Wang; Liping Guo; Guang Cheng; Chun Zhang; Shangbin Jin; Bien Tan; Andrew Cooper. Covalent Triazine Frameworks via a Low Temperature Polycondensation Approach. *Angew. Chem. Int. Ed.* **2017**, *56* (45), 14149–14153. https://doi.org/10.1002/anie.201708548.

(7) Manying Liu; Qi Huang; Shaolei Wang; Ziyong Li; Buyi Li; Shangbin Jin; Bien Tan. Crystalline Covalent Triazine Frameworks by In Situ Oxidation of Alcohols to Aldehyde Monomers. *Angew. Chem. Int. Ed.* **2018**, *57* (37), 11968–11972. https://doi.org/10.1002/anie.201806664.

(8) Matthias Georg Schwab; Birgit Fassbender; Hans Wolfgang Spiess; Arne Thomas; Xinliang Feng; Klaus Müllen. Catalyst-Free Preparation of Melamine-Based Microporous Polymer Networks through Schiff Base Chemistry. *J. Am. Chem.*

Soc. **2009**, *131* (21), 7216–7217. https://doi.org/10.1021/ja902116f.
(9) Song, W.-C.; Xu, X.-K.; Chen, Q.; Zhuang, Z.-Z.; Bu, X.-H. Nitrogen-Rich Diaminotriazine-Based Porous Organic Polymers for Small Gas Storage and Selective Uptake. *Polym. Chem.* **2013**, *4* (17), 4690–4696. https://doi.org/10.1039/C3PY00590A.
(10) Pillaiyar Puthiaraj; Kasi Pitchumani. Triazine-Based Mesoporous Covalent Imine Polymers as Solid Supports for Copper-Mediated Chan–Lam Cross-Coupling N-Arylation Reactions. *Chem. Eur. J.* **2014**, *20* (28), 8761–8770. https://doi.org/10.1002/chem.201402365.
(11) Liu, W.; Su, Q.; Ju, P.; Guo, B.; Zhou, H.; Li, G.; Wu, Q. A Hydrazone-Based Covalent Organic Framework as an Efficient and Reusable Photocatalyst for the Cross-Dehydrogenative Coupling Reaction of N-Aryltetrahydroisoquinolines. *ChemSusChem* **2017**, *10* (4), 664–669. https://doi.org/10.1002/cssc.201601702.
(12) Xiong, S.; Fu, X.; Xiang, L.; Yu, G.; Guan, J.; Wang, Z.; Du, Y.; Xiong, X.; Pan, C. Liquid Acid-Catalysed Fabrication of Nanoporous 1,3,5-Triazine Frameworks with Efficient and Selective CO_2 Uptake. *Polym. Chem.* **2014**, *5* (10), 3424–3431. https://doi.org/10.1039/C3PY01471A.
(13) Zhao, H.; Jin, Z.; Su, H.; Jing, X.; Sun, F.; Zhu, G. Targeted Synthesis of a 2D Ordered Porous Organic Framework for Drug Release. *Chem. Commun.* **2011**, *47* (22), 6389–6391. https://doi.org/10.1039/C1CC00084E.
(14) Ren, S.; Dawson, R.; Laybourn, A.; Jiang, J.; Khimyak, Y.; Adams, D. J.; Cooper, A. I. Functional Conjugated Microporous Polymers: From 1,3,5-Benzene to 1,3,5-Triazine. *Polym. Chem.* **2012**, *3* (4), 928–934. https://doi.org/10.1039/C2PY00585A.
(15) Zhonghua Xiang; Dapeng Cao. Synthesis of Luminescent Covalent–Organic Polymers for Detecting Nitroaromatic Explosives and Small Organic Molecules - Xiang. *Macromol. Rapid Commun.* **2012**, *33* (14), 1184–1190. https://doi.org/10.1002/marc.201100865.
(16) Xiang, Z.; Cao, D.; Huang, L.; Shui, J.; Wang, M.; Dai, L. Nitrogen-Doped Holey Graphitic Carbon from 2D Covalent Organic Polymers for Oxygen Reduction. *Adv. Mater.* **2014**, *26* (20), 3315–3320. https://doi.org/10.1002/adma.201306328.
(17) Mario R. Liebl; Jürgen Senker. Microporous Functionalized Triazine-Based Polyimides with High CO_2 Capture Capacity. *Chem. Mater.* **2013**, *25* (6), 970–980. https://doi.org/10.1021/cm4000894.
